# Ultrasound-guided placement of a midline catheter in a patient with extensive postburn contractures: A Case report

**DOI:** 10.1097/MD.0000000000014208

**Published:** 2019-01-18

**Authors:** Taisuke Yokota, Joho Tokumine, Alan Kawarai Lefor, Ayako Hasegawa, Tomoko Yorozu, Takayuki Asao

**Affiliations:** a Department of Anesthesiology, Kyorin University School of Medicine, Tokyo; b Department of Surgery, Jichi Medical University, Tochigi-ken; c Big Data Center for Integrative Analysis, Gunma University Initiative for Advanced Research, Maebashi, Gunma, Japan.

**Keywords:** burn, central venous catheter, midline, peripherally inserted central venous catheter, scar contracture

## Abstract

**Rationale:**

Obtaining venous access in a patient with extensive postburn scar contractures is a challenge.

**Patient concerns:**

A 39-year-old woman suffered a burn 2 years previously with a total body surface area burn of 93%, and a burn index of 85. Reconstructive surgery was previously performed 39 times. Split-thickness skin grafting to the neck was planned. She had no accessible peripheral veins.

**Diagnosis:**

Difficult venous access due to excessive burn scar contractures.

**Interventions:**

Central venous catheterization was considered impossible even with ultrasound guidance. We placed a midline catheter for intraoperative venous access in a patient with extensive burn scar contractures. The midline catheter is a peripheral venous catheter placed in an arm vein.

**Outcomes:**

We successfully placed a midline catheter in the right brachial vein. This catheter was used for 24 days without difficulty.

**Lessons:**

The midline catheter is a viable choice in patients with difficult vascular access due to extensive postburn scar contractures.

## 1 Introduction

To care for patients with extensive burns, venous access is needed for fluid and drug administration, and blood transfusions.^[1]^ Adequate vascular access in the operating room is essential and placing an indwelling catheter can be a challenge for the anesthesiologist. In the early resuscitation and long-term management phases in the care of patients with extensive burns, central venous catheters and peripherally inserted central catheters, allowing high flow rates and delivery of large fluid volumes, are often used.^[1]^ In the later phases of management, such as reconstructive surgery, any route for venous access, including routine peripheral venous catheters, is considered.

Midline catheters are defined as peripherally inserted catheters, which enter the venous circulation in the antecubital fossa or arm, about 8 to 20 cm in length and do not pass the axilla to become “centrally located.”^[2]^ We inserted a midline catheter in the patient's brachial vein under ultrasound guidance for the administration of anesthetic agents and intraoperative support during reconstructive surgery. We report successful use of the midline catheter in this patient with extensive postburn scar contractures.

### 1.1 Consent statement

Written informed consent was obtained from the patient for the publication of this case report.

## 2 Case report

A 39-year-old woman was scheduled to undergo split thickness skin grafts as part of a reconstructive program following extensive burns. She was burned in a house fire 2 years previously, when she suffered 93% total body surface area burns, with a burn index of 85. Her history was significant for having undergone 39 reconstructive operations over 2 years. Her neck was reconstructed using a graft from the latissimus dorsi, and both femoral veins were occluded due to multiple accesses and indwelling catheters. A subclavian venous catheter had been inserted once before under ultrasound guidance. Physical exam showed her weight was 49 kg, and her height was 155 cm. Most of her body was covered by hard contracted skin. There were no peripheral veins evident on inspection.

To obtain venous access for this operation, we searched for veins using a linear probe ultrasound device (6–15 MHz, SonoSite Edge, FUJIFILM SonoSite, Inc., Washington, USA) on the chest and arm. This revealed accessible veins, including the subclavian and axillary veins on the anterior chest, and the brachial vein of the right arm. An indwelling catheter in the right brachial vein was considered as the first choice to avoid possible complications of using the subclavian or axillary veins such as pneumothorax or hemothorax. The right brachial vein measured 3 mm by ultrasound imaging.

**Figure 1:**
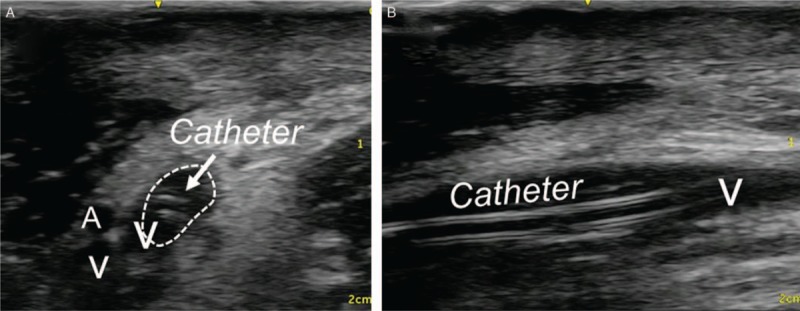
Ultrasound imaging after placement of the catheter. (A) Transverse view of the brachial vein. Dashed circle indicates the brachial vein. Arrow indicates the catheter inside the brachial vein. (B) Longitudinal view of the brachial vein. A catheter is present inside the brachial vein. Catheter = midline, V = brachial vein, A = brachial artery.

**Figure 2:**
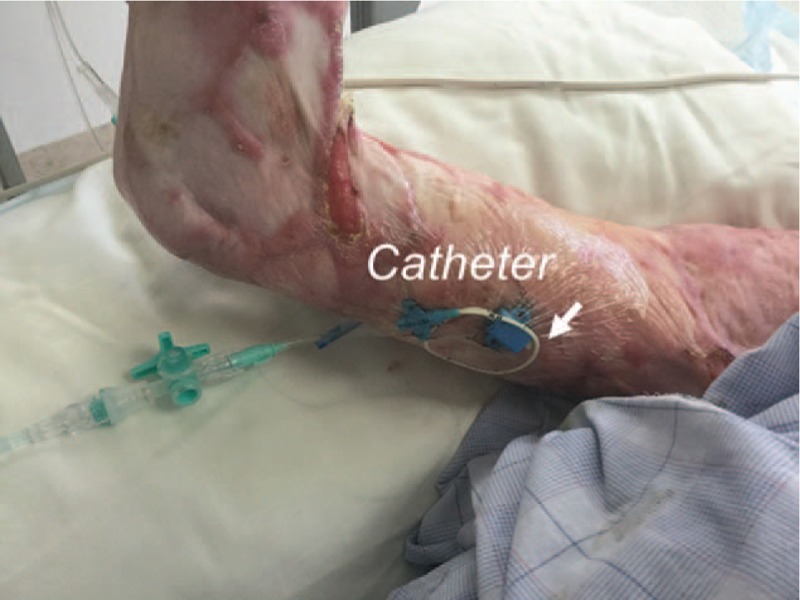
Midline catheter placed in the right arm. Arrow indicates skin insertion site in the arm.

**Figure 3:**
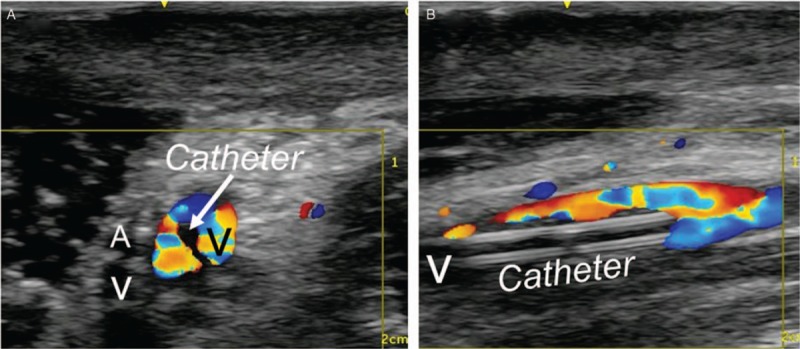
Blood flow in the vein after catheter placement. (A) Transverse view of the brachial vein. Mosaic pattern can be seen inside the brachial vein. Arrow indicates the catheter inside the brachial vein. (B) Longitudinal view of the brachial vein. The midline catheter is inside the brachial vein. Catheter = midline, V = brachial vein, A = brachial artery.

The right arm had restricted motion range due to extensive contractures. She was positioned in the right semilateral position to gain easy access to the medial side of the arm by supination and abduction. A pillow was placed behind her back to maintain this position. The skin of the arm was prepped with 1% chlorhexidine alcohol solution and covered with a sterile drape. The ultrasound probe was covered with a sterile plastic probe cover. The indwelling venous catheter was placed using sterile barrier precautions. Local anesthetic (5 mL of 1% lidocaine) was injected, and a 20G catheter-over-the needle (48 mm) was inserted. Ultrasound-guided venous catheterization was performed. To access the brachial vein, we used the short-axis out-of-plane approach to avoid mechanical complications of unanticipated artery or peripheral nerve injuries, and then used the long-axis in-plane approach to penetrate the anterior vein wall and cannulate the vein. After inserting the cannula, a guide wire was placed through the cannula using the modified Seldinger technique. We placed a single lumen polyurethane catheter (3 Fr., SMAC Plus, Nippon Covidien, Japan), a commercially available central venous catheter (there is no commercially available midline catheter in Japan). Successful insertion of the catheter was confirmed with ultrasound imaging (Fig. 1). The catheter entered the vein 6 cm from the skin entry site (Fig. 2). After placing the catheter, we checked venous flow using color Doppler imaging (Fig. 3).

Surgery was performed the following day with no adverse events. A split-thickness skin graft was harvested from the head, and grafted to the neck. The skin graft became infected several days later and the midline catheter was used for administration of antibiotics. A second skin graft was performed again postadmission day 16 and the catheter removed on postadmission day 24. She was discharged without complications.

## 3 Discussion

Venous access in patients with severe burn injuries is essential for resuscitation and perioperative care. Venous access in these patients is usually obtained using routine central venous catheters. The peripherally inserted central catheter is another option to obtain venous access in patients with burns.^[3,4]^ Routine peripheral venous catheters may be used, but the veins often become thrombosed and the catheters must be moved. Eventually in many patients, an adequate peripheral vein can no longer be identified for catheter placement.

The present patient had extensive burn scars. There were no obvious sites for peripheral venous access. She had undergone multiple operations and venous access was challenging each time. Internal jugular vein catheterization could not be performed because of the scarred skin. The femoral vein was occluded due to multiple previous access attempts. The subclavian vein was technically difficult, and carried the risk of hemothorax.^[5]^ If that complication did occur, we would have difficulty infusing adequate fluids for resuscitation.

A peripherally inserted central catheter may be used instead of a central venous catheter, with complication rates reportedly similar to central venous catheters in patients with burn injuries.^[1]^ However, the rate of mechanical complications during placement of a peripherally inserted central catheter is clearly lower than when placing a central venous catheter. We considered placing a peripherally inserted central catheter in an arm vein. This option was ruled out because of her inability to abduct the arm. A peripherally inserted central catheter may not pass the axilla, even if it could be inserted into an arm vein. Therefore, we chose a midline catheter for venous access. Recently, the clinical efficacy of the midline catheter for initial treatment of patients with burns was reported.^[6,7]^ This is the first report using a midline catheter for difficult vascular access in a patient with extensive postburn scar contractures.

The Michigan Appropriateness Guide for Intravenous Catheters (MAGIC) suggests that a midline catheter is a useful alternative for venous access instead of a peripherally inserted central catheter.^[8]^ The midline catheter can be used for an intermediate term compared to the limited short-term use of a peripheral catheter and the long-term use of a peripherally inserted central catheter or central venous catheter. Usually, the midline catheter is recommended to be used from 6 to 14 days.^[2]^ Use of the midline catheter has been reported up to 28 days.^[7]^ We used the midline catheter in the present patient for 24 days.

The midline catheter can be placed as a peripherally inserted catheter from the antecubital fossa or arm for a distance of 8 to 20 cm and not to go past the axilla.^[2]^ Recently, insertion of a midline catheter is recommended to be performed under ultrasound guidance and placing it in the arm. Placing it in the arm may limit the incidence of venous thrombosis.^[9]^ Usually, arm veins, especially the basilic and brachial veins have a larger caliber compared to antecubital fossa veins. Catheters placed in large caliber veins are associated with a lower risk of venous thrombosis.^[10]^ Placing the midline catheter in an arm vein should be associated with a lower incidence of venous thrombosis compared to a catheter placed in an antecubital fossa vein. In this patient, venous blood flow was maintained after placing the midline catheter.

The midline catheter has the advantage of a low risk for developing catheter-related blood stream infections.^[2,11]^ In this patient, the midline catheter was used for 24 days with no evidence of infectious complications. A midline catheter may be useful to gain venous access in patients with otherwise difficult access, such as a patient with extensive postburn contracture scars.

## Acknowledgments

The operator inserting midline was trained in a vascular access seminar, which was supported by AMED under Grant Number JP15km0908001.

## Author contributions

**Conceptualization:** Joho Tokumine, Takayuki Asao.

**Funding acquisition:** Takayuki Asao.

**Methodology:** Joho Tokumine.

**Project administration:** Joho Tokumine, Ayako Hasegawa.

**Validation:** Tomoko Yorozu.

**Visualization:** Tomoko Yorozu, Takayuki Asao.

**Writing – original draft:** Taisuke Yokota, Joho Tokumine.

**Writing – review & editing:** Alan Kawarai Lefor.

Joho Tokumine orcid: 0000-0003-3481-2085.
